# Efficient progerin clearance through autophagy induction and SRSF-1 downregulation in Hutchinson-Gilford Progeria Syndrome

**DOI:** 10.1186/1750-1172-10-S2-O9

**Published:** 2015-11-11

**Authors:** Karim Harhouri

**Affiliations:** 1 Aix Marseille Université, INSERM, GMGF UMR_S 910, 13385, Marseille, France

## 

Hutchinson-Gilford progeria syndrome (HGPS) is an extremely rare premature and accelerated aging disease caused by a de novo point mutation in *LMNA* encoding A-type lamins. Progerin, a truncated and toxic form of prelamin A, accumulates in HGPS cells nuclei and is a hallmark of the disease. We show that progerin is sequestered, together with other proteins (lamins B1/B2, emerin), into abnormally shaped nuclear organelles, identified as novel biomarkers in Progeria. We identified a novel compound that led to effective progerin degradation and clearance from patients’ fibroblasts. This compound induces progerin nucleocytoplasmic translocation, and progerin degradation through macroautophagy. It also strongly reduces progerin production through caspase-linked cleavage of SRSF-1 controlling prelamin A mRNA splicing. *In vivo*, upon treatment with the compound, progerin expression decreases in skeletal muscle of *Lmna^G609G/G609G^* mice. Altogether, we demonstrate increased progerin clearance based on the dual action of a novel compound and shed light on a novel promising class of molecules towards a therapy for Progeria and related diseases.

